# Definitions and adverse outcomes of sarcopenia in older patients in orthopedic and trauma care: A scoping review on current evidence

**DOI:** 10.1007/s00068-024-02541-8

**Published:** 2024-05-08

**Authors:** Jan Gewiess, Sebastian Kreuzer, Anna Katharina Eggimann, Dominic Bertschi, Johannes Dominik Bastian

**Affiliations:** 1 Department of Orthopaedic Surgery and Traumatology, Inselspital, Bern University Hospital, University of Bern, 3010 Bern, Switzerland; 2 Department of Geriatrics, Inselspital, Bern University Hospital, University of Bern, 3010 Bern, Switzerland

**Keywords:** Fracture, Older adults, Hospitalized, Muscle health, Functional decline, Mortality

## Abstract

**Purpose:**

Sarcopenia, defined as the loss of muscle mass and strength, can hinder postoperative recovery and raise mortality rates. However, the current evidence on the harmful effects of sarcopenia in older patients in orthopedic and trauma care is unclear. This scoping review investigates different definitions that were used for the diagnosis of sarcopenia in older patients in orthopedic and trauma care and what adverse consequences have been examined.

**Methods:**

We performed a comprehensive literature search in PubMed and Embase, following the PRISMA guidelines. We included original studies that examined clinical outcomes (such as length of hospital stay, rate of non-home discharge, rate of subsequent falls, rate of refractures, mortality, and functional outcome/quality of life) in older patients in orthopedic and trauma care (aged 65 years and above) with diagnosed sarcopenia (S) compared to a group without sarcopenia (NS).

**Results:**

Our search identified 2,748 publications. Out of these, 23 articles met the inclusion criteria. Most publications were from Asia (n = 13). A total of 6174 patients were examined, with a prevalence of sarcopenia in 14–92%. 11 articles focused on patients with hip joint pathologies. Most studies diagnosed sarcopenia according to the Asian Working Group on Sarcopenia (AWGSOP1 or AWGSOP2) definitions (n = 10). Length of hospital stay was investigated in 13 studies. Seven studies assessed rates of non-home discharge rates. Subsequent falls were not investigated in any of the studies. 1 study reported the overall refracture rate (S: 10.4%; NS: 5.8%). Mortality was assessed in 11 studies (S: 1–60.5%; NS: 0–39.5%). The functional outcome/quality of life was investigated by 17 studies (Barthel Index decline S: -4.5 to -15.3 points; NS: -11.7 to -54.7 points).

**Conclusion:**

Sarcopenia has been increasingly studied in older patients in orthopedic and trauma care but there is a lack of consistent definition criteria. This scoping review suggests that sarcopenia may be associated with prolonged length of stay, higher rates of non-home discharge, and increased mortality among older patients in orthopedic and trauma care. However, prospective studies are necessary to establish the relationship between sarcopenia and refractures, falls, and functional outcome/quality of life among older patients in orthopedic and trauma care.

## Introduction

Sarcopenia is characterized by a decline of muscle mass and strength and is prevalent in 10–27% of individuals over 60 years old [1]. Despite the heightened interest in sarcopenia research, as illustrated by the annual increase of 18% in the global literature since 2000, the diagnosis of sarcopenia is still highly inconsistent and underestimated [2, 3]. At regular intervals, international consortia have proposed and updated different definitions and reference values of sarcopenia for American (Foundation for the National Institutes of Health, FNIH1/2), Asian (Asian working group for sarcopenia, AWGS1/2), and European (European working group for sarcopenia, EWGSOP1/2) populations (Table 1) [4–6]. However, the fundamental criterion shared among these definitions is the presence of both low muscle mass (e.g., skeletal muscle mass assessed using bioelectric impedance analysis) and strength (e.g., handgrip strength assessed using a dynamometer). A recent systematic review found that orthopedic patients with low muscle mass and strength experience hindered recovery and heightened postoperative mortality, particularly in cases requiring emergency surgery [7]. Concordantly, in the orthogeriatric context, sarcopenia has been recently shown to be a univariate predictor of survival [8]. Yet, the global evidence regarding functional decline, rates of institutionalizations, subsequent falls, fractures, and mortality specifically in geriatric patients in orthopedic and trauma care suffering from sarcopenia is currently unclear. Consequentially, the purpose of our study was to conduct a scoping review to investigate the diagnostic definitions and adverse outcomes of sarcopenia, specifically in the field of orthogeriatrics.

**Table 1:** Diagnostic criteria for sarcopenia based on working group consensus definitions

Sarcopenia consensus definition	Low muscle strength (grip strength)		Low muscle mass (appendicular skeletal muscle mass/height ratio)			Low muscle performance (gait speed)	
	Women	Men	Women	Men		Women	Men
AWGS1	(< 18 kg)	(< 26 kg)	< 5.4 kg/m^2^	< 7.0 kg/m^2^		(< 0.8 m/s)	(< 0.8 m/s)
AWGS2	< 18 kg	< 28 kg	< 5.4 kg/m^2^	< 7.0 kg/m^2^			
EWGSOP1	< 20 kg	< 30 kg	(< 5.5 kg/m^2^)	(< 7.26 kg/m^2^)		< 0.8 m/s or SPPB < 8 pts	< 0.8 m/s or SPPB < 8 pts
EWGSOP2	< 16 kg or chair rise test < 15 s	< 27 kg or chair rise test < 15 s	< 5.5 kg/m^2^	< 7.0 kg/m^2^			
FNIH1			< 0.512 kg/kg/m^2^	< 0.789 kg/kg/m^2^			
FNIH2	< 16 kg	< 26 kg	< 0.512 kg/kg/m^2^	< 0.789 kg/kg/m^2^			

Facultative measures in the respective guideline are displayed in parentheses; SPPB—short physical performance battery

## Methods

Prior to undertaking this review, an a priori research protocol was developed. The scoping review was performed according to the ‘Preferred Reporting Items for Systematic Reviews and Meta-Analyses extension for Scoping Reviews guidelines’ (PRISMA-SCr) [9]. Two reviewers (JG and SK) independently screened the literature in a systematic approach to filter relevant articles for this scoping review. Discrepancies were resolved by discussion and involvement of a third party where necessary.

The databases of PubMed and Embase were searched using the dedicated search string ‘sarcopen* AND orthop* OR sarcopen* AND fracture’ up to 2023–08-20. We included original articles investigating patients aged ≥ 65 years (acutely) hospitalized with an orthopedic (trauma) diagnosis. We included studies with and without orthogeriatric co-management. Included articles had to report on at least one sarcopenic (sub-) group and one control group. A diagnostic definition of sarcopenia had to be outlined. Articles were included if they investigated adverse outcomes associated to sarcopenia according to the paper by Stuck AK et al. [10] including length of hospital stay, the rate of non-home discharge (nursing home, long-term care), the rate of subsequent falls, the rate of subsequent refractures, functional outcome/quality of life, or mortality. Only original articles published in English, French, or German were considered in this review.

Reviews, meta-analyses, animal studies, editorials, comments, and letters were excluded as well as studies in languages other than English, French, or German. Studies investigating cohorts with specific underlying diseases such as malignancy, rheumatoid arthritis, M. Parkinson, or HIV were also excluded.

Data was extracted from full-text assessment and from published supplemental material whenever available. Data extraction covered information on the study population (origin of the study, existence of orthogeriatric co-management, injury localization, demographic parameters), definition criteria for sarcopenia (methods and reference values), and outcome parameters (length of hospital stay, rate of non-home discharge, rate of subsequent falls, rate of refractures, mortality, functional outcome/quality of life).

Statistical analyses and graphing were performed using ‘R’ (R Core Team, Version 4.1.0; R Foundation for Statistical Computing, Vienna, Austria) and GraphPad Prism (Version 9.5 (2023); GraphPad Software, La Jolla, CA, USA). Descriptive statistics were obtained for relevant items. Odds ratios and 95% confidence intervals (CI) were calculated for sarcopenic and non-sarcopenic subgroups.

## Results

### Literature search and study characteristics

**Fig. 1:**
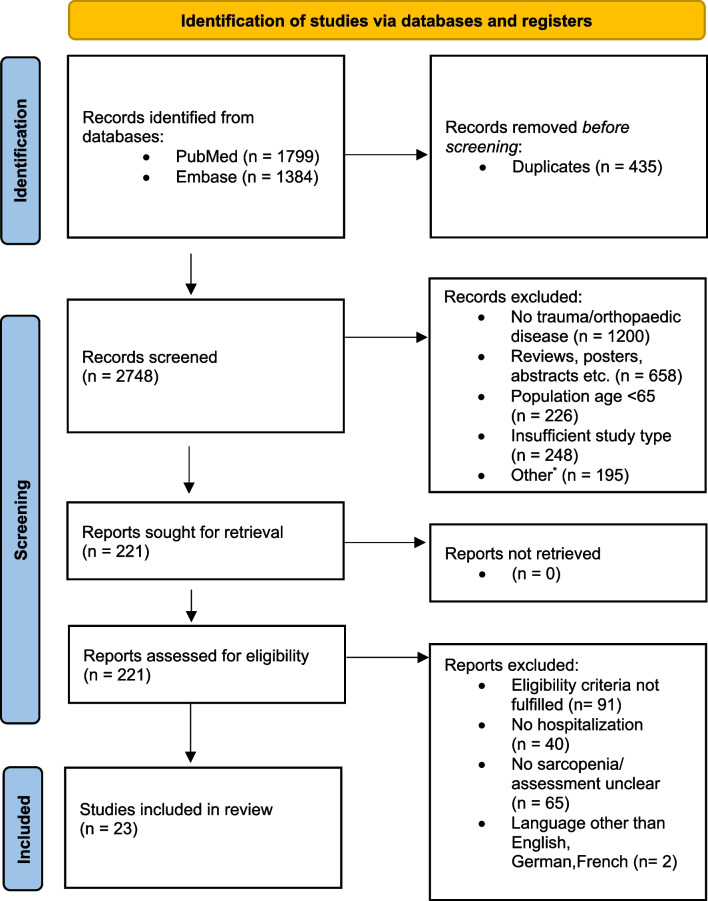
Flow diagram indicating the literature identification pathway resulting in the inclusion of 23 original articles according to the ‘Preferred Reporting Items for Systematic Reviews and Meta-Analyses’(PRISMA) guidelines. *—Investigations into sarcopenia within cohorts affected by specific underlying diseases (e.g., malignancy, rheumatoid arthritis, Parkinson's disease, HIV), studies in basic science, and studies that were not pertinent to the research inquiries

Our literature search identified 3183 records. After duplicate removal, 2748 records were screened. Of these, 221 full-text articles were assessed for eligibility leaving 23 studies with a cumulative number of 6174 patients included in this scoping review (Fig. 1). Included study types were 10 prospective and 13 retrospective case control, cohort, and cross-sectional studies. Three studies reported an orthogeriatric co-management. All studies were published between 2016 and 2020. Follow up time of included studies ranged from 6 to 48 months.

### Study populations

**Fig. 2:**
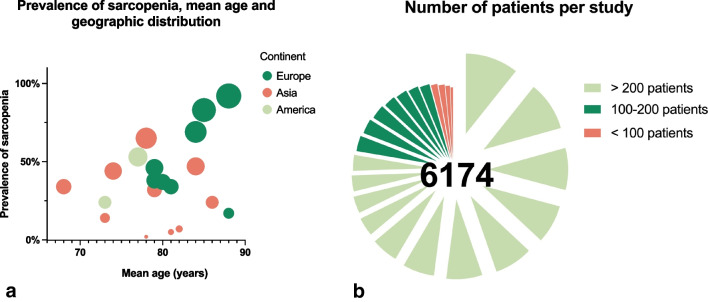
**a** Bubble diagram depicting the demographic characteristics of the articles included in this scoping review. Most studies originated from Asia and Europe. **b** Pie diagram showing cohort sizes of included studies

Studies investigating adverse outcomes associated to sarcopenia in orthopedics primarily originated from Asia (n = 13), followed by Europe (n = 8) and the US (n = 2) (Fig. 2a). Cohort sizes ranged between 36 and 661 patients (Fig. 2b). The mean age of the underlying populations ranged between 68 and 88 years. In 20 studies, a predominantly female population was investigated (54–80%). Nine studies recorded the pre-hospitalization residential status (0–29% institutionalized before hospital admission). The injury/disease localization was predominantly the hip region (n = 11 studies), followed by the spine (n = 8) (Table 2). Most studies evaluated traumatic injuries (n = 17). Patients undergoing elective surgery without prior trauma were investigated in five studies in knee and spine patients, respectively.

**Table 2:** Demographic characteristics of the articles included in this scoping review

Author, year of publication	Country	Number of participants	Mean age (years)	Female sex	Institutionalized residential status*	Injury localization	Trauma/elective	Working Group definition
Bermejo-Bescós, Martín-Aragón et al. 2020 [11]	Spain	150	88	79%	22%	hip	trauma	EWGSOP1
Chen, Lei et al. 2022 [12]	China	214	78	72%	unknown	spine	trauma	AWGS1
González-Montalvo, Alarcón et al. 2016 [13]	Spain	479	85	80%	22%	hip	trauma	EWGSOP1
He, Cai et al. 2021 [14]	China	525	68	68%	unknown	knee	elective	AWGS1
Iida, Sakai et al. 2018 [15]	Japan	396	82	72%	7%	spine	trauma	AWGS1
Iida, Seki et al. 2021 [16]	Japan	337	84	80%	29%	hip	trauma	AWGS1
Inose, Yamada et al. 2018 [17]	Japan	85	74	61%	unknown	spine	elective	AWGS1
Jiang, Ding et al. 2022 [18]	China	144	86	74%	22%	hip	trauma	EWGSOP2 and AWGS2
Kaplan, Pham et al. 2017 [19]	USA	450	77	40%	unknown	all	trauma	none
Kristensen, Hulsbæk et al. 2021 [20]	Denmark	182	79	75%	0%	hip	trauma	EWGSOP2 and FNIH2
Landi, Calvani et al. 2017 [21]	Italy	127	81	65%	unknown	hip	trauma	FNIH1
Liao, Chen et al. 2022 [22]	Taiwan	482	72	75%	unknown	knee	elective	AWGS2
Lim, Beom et al. 2019 [23]	Korea	80	81	78%	unknown	hip	trauma	AWGS1
Malafarina, Malafarina et al. 2019 [24]	Spain	187	85	74%	1%	hip	trauma	EWGSOP2
Ohyama, Hoshino et al. 2021 [25]	Japan	60	78	78%	unknown	spine	trauma	AWGS1
Pernik, Hicks et al. 2022 [26]	USA	196	73	54%	unknown	spine	elective	none
Sakai, Wakao et al. 2020 [27]	Japan	235	73	43%	unknown	spine	elective	AWGS1
Sim, Lee et al. 2021 [28]	Korea	615	81	72%	unknown	hip	trauma	none
Steihaug, Gjesdal et al. 2017 [29]	Norway	202	80	75%	0%	hip	trauma	EWGSOP1
Steihaug, Gjesdal et al. 2018 [30]	Norway	201	79	76%	0%	hip	trauma	EWGSOP1
Takahashi, Kubo et al. 2018 [31]	Japan	36	84	69%	unknown	spine	trauma	AWGS1
Toyoda, Hoshino et al. 2019 [32]	Japan	130	78	43%	unknown	spine	other	AWGS1
Wiedl, Förch et al. 2022 [8]	Germany	661	85	75%	26%	all	trauma	none

* e.g., living in nursing homes, supervised flats etc.

Abbreviations: AWGS – Asian Working Groupd for Sarcopenia; EWGSOP – European Working Group for Sarcopenia; FNIH – Foundation of the National Institutes of Health

### Diagnostic criteria of sarcopenia

**Fig. 3:**
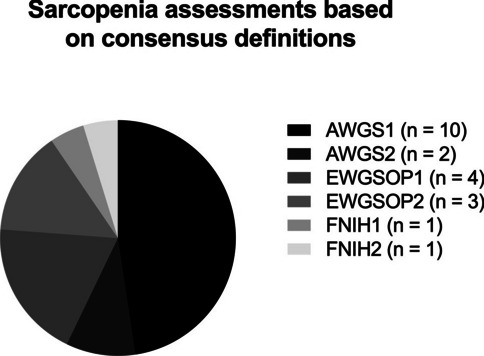
Frequency of sarcopenia assessments based on consensus definitions of the Asian Working Group for Sarcopenia (AWGS), European Working Group for Sarcopenia (EWGSOP) and the Foundation of the National Institutes of Health (FNIH) in patients in orthogeriatric care

Overall, the prevalence of sarcopenia ranged between 14 and 92% (Fig. 2a). Assessment criteria, methodological approaches, and reference values to diagnose sarcopenia varied between studies (Tables 1 and 2). Most studies (n = 10) defined sarcopenia relying on one single parameter, of which low muscle mass was the most used criterion (n = 6). Sarcopenia was defined according to the criteria of AWGS1 in 10 studies, followed by the EWGSOP1 (n = 4), EWGSOP2 (n = 3), and AWGSOP2 (n = 2; Fig. 3). Four studies did not adhere to the working group consensus criteria for diagnosing sarcopenia (Table 2).

### Length of stay

**Fig. 4:**
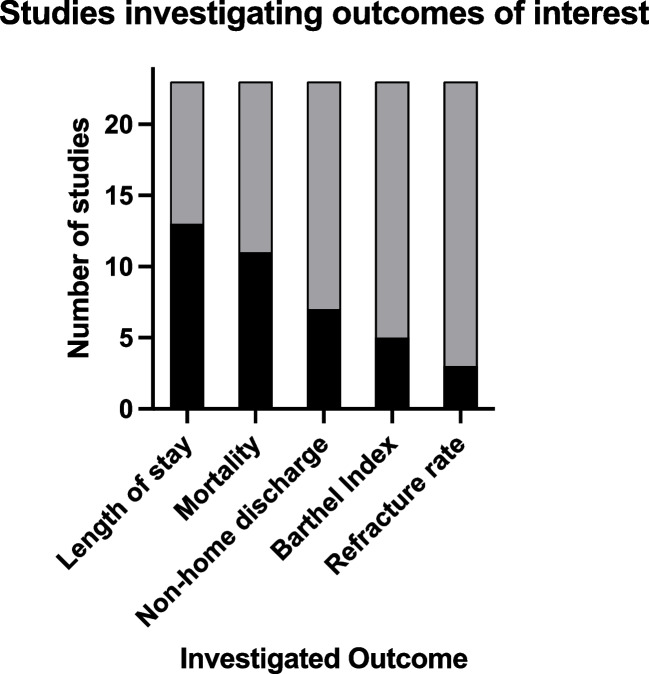
Number of studies investigating outcomes of interest among sarcopenic patients in orthogeriatric care

Length of hospital stay was evaluated in 13 studies (Fig. 4). The average length of stay ranged between 5.6 and 35.3 days in sarcopenic patients (Table 3). In the non-sarcopenic study groups, the average length of stay ranged between 5.2 and 32.5 days. Ten of 13 studies evaluating length of stay reported a trend towards a prolonged stay in the sarcopenic group compared to the non-sarcopenic group (Table 3). However, only four studies showed significant differences (p < 0.05).

**Table 3:** Adverse events and functional outcomes/quality of life in studies assessing sarcopenia in older patients in orthopedic and trauma care

Author, year of publication	Study type	Follow-up (months)	Sarcopenia prevalence	Length of hospital stay		Functional outcome/quality of life		Rate of refractures		Non-home discharge		Mortality	
				*Sarcopenia group (days)*	*Control group (days)*	*Sarcopenia group*	*Control group*	*Sarcopenia group*	*Control group*	*Sarcopenia group*	*Control group*	*Sarcopenia group*	*Control group*
Bermejo-Bescós, Martín-Aragón et al. 2020 [11]	prospective cohort study	12	0.920	not specified	not specified	not specified	not specified	not specified	not specified	not specified	not specified	0.12	0.11
Chen, Lei et al. 2022 [12]	retrospectiv case control study	Minimum 3	0.322	not specified	not specified	not specified	not specified	0.84	0.11	not specified	not specified	not specified	not specified
González-Montalvo, Alarcón et al. 2016 [13]	prospective cohort study	0.33	0.171	9.5 ± 3.7	10.32 ± 5.2	Barthel Index decrease: 59.5 ± 17.9	Barthel Index decrease: 54.7 ± 20.3	not specified	not specified	0.79	0.68	0.01	0.045
He, Cai et al. 2021 [14]	retrospective case control study	12	0.343	9.58 ± 2.26	9.5 ± 2.66	Gait Speed: 0.72 ± 0.22 m/s	Gait Speed: 0.93 ± 0.20 m/s	not specified	not specified	not specified	not specified	not specified	not specified
Iida, Sakai et al. 2018 [15]	prospective observational cohort study	12	0.699	not specified	not specified	Barthel Index decrease: 16.57 JOA: 8.84	Barthel Index decrease: 11.73 JOA: 8.56	not specified	not specified	0.690	0.538	not specified	not specified
Iida, Seki et al. 2021 [16]	retrospective case–control study	18	0.685	28.8 ± 18.6	28.3 ± 14.3	Barthel Index decrease: -19.6	Barthel Index decrease: -15.5	not specified	not specified	0.519	0.396	0.13	0
Inose, Yamada et al. 2018 [17]	retrospective cohort study	12	0.435	not specified	not specified	L-JOA increase: 8.2 VAS decrease: 33.1	L-JOA increase: 9.6 VAS decrease: 26.7	not specified	not specified	not specified	not specified	not specified	not specified
Jiang, Ding et al. 2022 [18]	retrospective cross sectional study	12	0.240	31.03	10.09	EQ-5D increase: 6.9	EQ-5D increase: 2.8	not specified	not specified	0.3428	0.174	0.2857	0.0826
Kaplan, Pham et al. 2017 [19]	retrospective cohort study	12	0.530	7	6.7	not specified	not specified	not specified	not specified	0.625	0.557	0.13	0.031
Kristensen, Hulsbæk et al. 2021 [20]	prospective observational cohort study	12	0.462	13.7	11.2	not specified	not specified	not specified	not specified	not specified	not specified	0.23	0.041
Landi, Calvani et al. 2017 [21]	prospective observational cohort study	3	0.339	30.8	29.4	not specified	not specified	not specified	not specified	not specified	not specified	not specified	not specified
Liao, Chen et al. 2022 [22]	retrospective observational cohort study	8	0.373	6.59	6.88	not specified	not specified	not specified	not specified	not specified	not specified	not specified	not specified
Lim, Beom et al. 2019 [23]	prospective observational cohort study	6	0.438	22.3 ± 5.7	20.5 ± 5.6	Recovery to prefracture walking ability: 0.615	Recovery to prefracture walking ability: 0.7	not specified	not specified	not specified	not specified	not specified	not specified
Malafarina, Malafarina et al. 2019 [24]	prospective observational cohort study	48	0.508	not specified	not specified	Barthel Index Decrease: 25.06 pts Gait Speed: 0.391 m/s	Barthel Index Decrease: 29.19 pts Gait Speed: 0.389 m/s	not specified	not specified	not specified	not specified	0.605	0.395
Ohyama, Hoshino et al. 2021 [25]	retrospective case control study	6	0.650	not specified	not specified	Physical Performance of SF-36 Increase: 22.7 VAS Reduction: 52.3	Physical Performance of SF-36 Increase: 21.93 VAS Reduction: 28.8	adjacent fracture: 0.385	adjacent fracture: 0.429	not specified	not specified	not specified	not specified
Pernik, Hicks et al. 2022 [26]	retrospective case series study	48	0.245	5.6	5.2	not specified	not specified	not specified	not specified	0.146	0.203	0.125	0.047
Sakai, Wakao et al. 2020 [27]	prospective case–control study	12	0.140	not specified	not specified	Gait Speed (m/s) preOP: 0.89 ± 0.52; postOP: 1.15 ± 0.32 EQ5D preOP: 0.47 ± 0.2; postOP: 0.64 ± 0.2 SF36 preOP: 35.33 ± 13.13; postOP: 49.12 ± 19.16	Gait Speed (m/s) preOP: 1.20 ± 0.48; postOP: 1.37 ± 0.69 EQ5D preOP: 0.57 ± 0.17; postOP: 0.78 ± 0.18 SF36 preOP: 41.81 ± 16.6; postOP: 63.13 ± 23.91	not specified	not specified	not specified	not specified	not specified	not specified
Sim, Lee et al. 2021 [28]	retrospectiv case control study	> 12	0.502	7	7	not specified	not specified	not specified	not specified	not specified	not specified	Overall 0.408 1 Year Mortality: 0.197	Overall 0.245 1 Year Mortality: 0.092
Steihaug, Gjesdal et al. 2017 [29]	prospective cross sectional study	0.25	0.366	7.5	6	NMS: 7 pts Barthel-Activities of Daily living: 20 pts	NMS: 9 pts Barthel-Activities of Daily living: 20 pts	not specified	not specified	not specified	not specified	not specified	not specified
Steihaug, Gjesdal et al. 2018 [30]	prospective observational cohort study	12	0.383	9.6 ± 6.7	6.8 ± 2.7	NMS change: -1.3 ± 1.9 B-ADL change: -2.2 ± 3.9	NMS change: -1.2 ± 1.8 B-ADL change: -0.8 ± 2.4	0.104	0.058	0.429	0.371	0.065	0.024
Takahashi, Kubo et al. 2018 [31]	retrospective cohort study	1	0.472	35.3 ± 16.3	32.5 ± 12.0	Barthel Index Increase: 47.3	Barthel Index Increase: 62.7	not specified	not specified	not specified	not specified	not specified	not specified
Toyoda, Hoshino et al. 2019 [32]	retrospective observational cohort study	40	0.200	not specified	not specified	JOA change: -11.2	JOA change: -29.1	not specified	not specified	not specified	not specified	not specified	not specified
Wiedl, Förch et al. 2022 [8]	retrospective case series study	24	0.832	not specified	not specified	not specified	not specified	not specified	not specified	not specified	not specified	0.459	0.29

* e.g., living in nursing homes, supervised flats etc.

Abbreviations: B-ADL – Bayer Activities of Daily Living Scale, JOA – Japanese Orthopaedic Association, NMS – New Mobility Score, VAS – Visual Analogue Scale

### Rate of non-home discharge

Seven studies assessed the discharge destination (Table 3). Non-home discharge (re- institutionalization to nursing home, rehabilitation unit or long-term care) was necessary in 12 to 79% of patients with sarcopenia with an odds ratio ranging between 1.1 and 3.2 compared to patients without sarcopenia (17–68% non-home discharge). Gonzalez-Montalvo et al. had an in-depth look at the discharge destination in older patients in orthopedic and trauma care sustaining hip fractures [13]. Prior to the injury, patients with sarcopenia were more likely to live in nursing homes. However, discharge destinations were similar and rates of re-institutionalization nursing home, rehabilitation unit, long-term care were high in both, sarcopenic and non-sarcopenic groups (77% and 68%, respectively).

### Rate of subsequent falls

Subsequent falls have not been assessed by any of the studies reviewed.

### Rate of refractures

The overall refracture rate was assessed in only one publication (Table 3). Among 201 sarcopenic patients with hip fractures, Steihaug et al. detected an overall refracture rate of 10% (OR: 1.9; CI 0.66 – 5.12) [30]. Two studies reported on specific refractures: Chen et al. reported on vertebral refractures among 214 sarcopenic patients after percutaneous kyphoplasty and found a vertebral refracture rate of 84% (OR: 42.5; 95%CI 18 – 95.2) [12]. In addition, Ohayama et al. investigated the risk of adjacent vertebral fractures in patients suffering an osteoporotic spinal fracture [25]. They report on a prevalence of 39% of adjacent vertebral fractures (OR: 0.83; CI: 0.29 – 2.5).

### Mortality

Seven of eleven publications assessing overall mortality reported on one-year mortality (Table 3) ranging between 1 and 46%. Corresponding Odds ratios ranged between 1 (0.5–1.4) and 6.87 (2.2–19.2) with a higher risk of one-year mortality for sarcopenic patients.

### Functional outcome and quality of life

The functional outcome/quality of life has been described in 14 articles (Table 3). The Barthel Index was the most frequently used score (n = 5). Compared to the non-sarcopenic groups, four articles reported decline of function in the sarcopenic groups (Barthel Index -4.5 to -15.3 points).

Besides the Barthel Index, a variety of patient related outcome measurements (PROMs) were examined in the included studies (Table 3): Two out of three studies examining gait speed demonstrated slower gait in their sarcopenic subgroups. All three studies assessing the Japanese Orthopaedic Association Score showed inferior results in their sarcopenic subgroups. Two studies evaluated the EQ-5D score, with one indicating poorer values in the sarcopenic subgroup. Similarly, two studies investigated the 36-Item Short Form Health Survey score, with one revealing lower values in the sarcopenic subgroup. One of the two studies investigating the New Mobility Score reported worse outcomes in the sarcopenic subgroup, while both studies noted a similar change in NMS from pre- to postoperatively. One study found no difference in Activities of Daily Living scores between sarcopenic and non-sarcopenic groups. Another study explored the rate of patients regaining prefracture walking ability and identified a lower rate in the sarcopenic subgroup.

## Discussion

This scoping review provides a reconnoitering mapping of the available original literature elaborating on clinical outcome categories in defined sarcopenic and orthogeriatic patients. Through our investigation, we have identified several research gaps related to geographic and demographic subpopulations, the assessment and definition of sarcopenia, and the adverse events associated with sarcopenia in orthogeriatrics.

To the best of our knowledge, this study is the first scoping review conducted to investigate the available evidence on the assessment of sarcopenia and comprehensive evaluation of adverse outcomes in older patients in orthopedic and trauma care who are hospitalized. Previous systematic reviews on sarcopenia in orthopedic patients have included younger patients and focused on mortality or postoperative functional recovery after hip, spine, or distal radius surgeries [7, 33–39].

More than half of the current body of research on sarcopenia in older patients in orthopedic and trauma care is derived from populations in eastern Asia. Thus, according to the origin of most of the studies included, it is not surprising that the assessment criteria and reference values of the AWGS were used in most of the studies included in this review.

The published working group consensus criteria exhibit notable discrepancies. For instance, the EWGSOP2 algorithm requires the presence of both low muscle strength and low muscle mass for a sarcopenia diagnosis, while the EWGSOP1 criteria consider either low muscle mass or low muscle strength. Moreover, there are variations in the reference values across the working group criteria. For example, in women, low muscle strength according to the FNIH2 criteria corresponds to only 80% of the low muscle strength defined by the EWGSOP1 criteria (Table 1). Consequently, Stuck et al. demonstrated that the prevalence of sarcopenia varies significantly (1%—17%) in a cohort of 1495 community-dwelling participants based on the sarcopenia definition employed [40]. Similarly, Kim et al. reported a disparity in sarcopenia prevalence (2%—28%) depending on the underlying definition [41]. As highlighted by Bischoff-Ferrari et al., these discrepancies in prevalence may introduce biases in causal relationship analyses, as different sarcopenia definitions do not equally predict sarcopenia-related complications [42]. Considering the diverse age groups, procedural interventions (trauma vs. elective cases), anatomical regions assessed, and the geographic diversity of study populations, the heterogeneous nature of sarcopenia diagnostics precludes a robust meta-analysis.

Interestingly, despite well-established elements for sarcopenia assessments, four studies did not use such consensus criteria. Instead, three studies calculated a psoas vertebral muscle index. The ratio of patients identified with sarcopenia in these studies was similar compared to those using consensus criteria (24–51%). In their study of 450 older trauma patients, Kaplan et al. used the total cross-sectional psoas muscle area at the L3 level and used reference values to define sarcopenia (male < 52.4 cm^2^/m^2^; female < 38.6 cm^2^/m^2^) [19]. Similarly, among 196 spine patients, Pernik et al. assessed a psoas lumbar vertebral index (cross-sectional area of the psoas muscle on axial CT images at L3 versus area of the L3 vertebra on axial CT images)), based on which patients were divided into quartiles [26]. The patients ranging in the lowest psoas lumbar vertebral index quartile were defined as sarcopenic. Sim et al. used a psoas lumbar vertebral index at the L4 level and assigned patients according to the median to a ‘low’ or ‘high’ group among 615 patients with hip fractures [28]. Wiedl et al. measured the maximum calf circumference and defined 83% of their patients with less than 33 cm calf circumference as sarcopenic [8]. In summary, the heterogeneity of the underlying sarcopenia definitions precludes from a meta-analysis of outcome parameters.

Previous studies have demonstrated a clear association between sarcopenia and prolonged length of hospital stay, particularly in individuals below the age of 65 [43]. Our review indicates a potential trend towards prolonged hospital stays in orthogeriatric patients with sarcopenia. Nevertheless, the clinical relevance of this extension is uncertain, given that only four studies reported statistically significant findings and most of the differences were only marginal. Only Gonzalez-Montalvo et al. found a slightly shorter hospital stay in sarcopenic patients with hip fractures compared to non-sarcopenic patients [13]. However, this study was conducted in a specialized orthogeriatric unit providing a high standard of care including nutrition and physical exercise aimed at reducing length of hospital stay and may therefore not be comparable to studies conducted in other environments.

Non-home discharge may reflect negative outcomes such as declining overall health, which can be caused by conditions like delirium, dementia, or secondary diseases such as pneumonia. In addition, it may be secondary to (surgical) treatment failure, inadequate pain management, and the inability to walk independently. The potential link between institutionalization and sarcopenia in geriatric patients receiving orthopedic and trauma care could not be substantiated in this scoping review as the literature indicated high rates of re-institutionalization to nursing homes, rehabilitation units, and long-term care for both sarcopenic and non-sarcopenic groups (77% and 68%, respectively).However, in the orthogeriatric population specifically, the acute hospitalization may simply reveal an existing inability to live independently for both patients and their families. Therefore, non-home discharge may be seen as a symptom associated with the progression of sarcopenia rather than a direct consequence.

In their systematic review on sarcopenia and its correlation with falls and fractures in older adults, Yeung et al. found a heightened risk of falls in sarcopenic individuals (OR 1.60; 95%CI 1.37–1.86) [39]. While their study encompassed older adults without a lower age limit but with a mean or median age of over 65 years, it did not specifically focus on patient populations undergoing orthopedic or trauma surgeries. In the context of orthopedic and trauma care, patients are often instructed to adhere to postoperative weight-bearing restrictions, sometimes necessitating the use of assistive devices for walking. Despite efforts by orthopedic and trauma surgeons to minimize these restrictions in older patients, they may be unavoidable in certain cases. In addition to factors such as coordination ability, cognitive function, and physical decline, older patients undergoing orthopedic and trauma care may have to significantly higher rates of falls and subsequent fractures compared to non-orthopedic patient populations.

Refracture rates within a time frame of 12 to 36 months following hip fracture have been documented to vary between 6 and 15% [44]. The observed 10% overall refracture rate after hip fractures among sarcopenic older patients in orthopedic and trauma care does not indicate an elevated refracture risk in this population [13]. After percutaneous vertebroplasty of spine fractures, there is a possibility of spinal refractures occurring in up to 52% of patients within a period of 7 years [45]. Chen et al. conducted a multivariate analysis to account for confounding variables and determined that sarcopenia was associated with a significantly increased risk of spinal refracture following kyphoplasty, with a prevalence rate of 84% [12]. They attribute this finding to the reduction in stability and the increase in pressure on the vertebral body as a result of muscle atrophy and degeneration associated with sarcopenia.

Multiple systematic reviews portray sufficient evidence regarding the association of sarcopenia and mortality in orthopedic patients [7, 34, 37]. Our scoping review suggests such a similarly consistent association between sarcopenia and mortality in the orthogeriatric population. The wide range of the published 1-year mortality rates (1–73%) may be secondary to the respective studies’ study populations and sarcopenia assessment criteria. Such heterogeneity precludes from a meta-analysis based on the existing literature.

Using various tools (patient related outcome measurement, PROMs), many studies evaluated the quality of life among their patients. Our review suggests that sarcopenia is associated with a decreased general functional outcome and quality of life compared to non-sarcopenic orthogeriatric populations. However, it is important to note that the heterogeneity of the PROMs used in these studies makes it difficult to determine the exact extent of this association.

Our scoping review reveals that the rates of refractures and subsequent falls have been insufficiently investigated in older patients in orthopedic and trauma care. The possible association of refractures and subsequent falls to sarcopenia can neither be expected nor excluded.

One major drawback of the literature review approach is always the potential for incomplete retrieval of relevant studies. However, this risk is mitigated by the involvement of two independent reviewers who conducted the systematic literature screening. Further limitations of this review’s methodology include language and age restrictions. This may explain some of the low variation regarding the geographic distribution of the included studies. Although we only included studies investigating patients over the age of 65 years, the mean age varied significantly among the different studies. This may be an important confounder regarding some of the results presented in our review. However, by exclusively including patients aged 65 years and above, we ensure a specific focus on the orthogeriatric population. In conclusion, caution should be exercised in all interpretations and deductions due to the heterogeneity of study populations, sarcopenia definitions, injury and disease modalities and localizations, follow-up periods, and outcome assessment tools discussed in this review.

## Conclusion

This scoping review found that sarcopenia has been increasingly studied in older patients in orthopedic and trauma care. However, various definitions of sarcopenia were applied not permitting pooling of data in a meta-analytical approach. In summary, there is an ongoing need for an international consensus on a universally accepted definition of sarcopenia. This scoping review suggests that sarcopenia may be linked to greater length of hospital stay, higher rates of non-home discharge, and increased mortality. In specific, prospective studies should be conducted in order to understand the causal relationship between sarcopenia and refractures, falls, and functional outcome/quality of life among older patients in orthopedic and trauma care. Additionally, the establishment of large registers might help to identify patients at increased risk for negative outcomes and contribute to implement preventive measures.
